# Wavelength-swept spontaneous Raman spectroscopy system improves fiber-based collection efficiency for whole brain tissue classification

**DOI:** 10.1117/1.NPh.11.2.025007

**Published:** 2024-06-19

**Authors:** Elahe Parham, Antoine Rousseau, Mireille Quémener, Martin Parent, Daniel C. Côté

**Affiliations:** a CERVO Brain Research Center, Québec City, Québec, Canada; b Université Laval, Centre d’optique, photonique et laser, Québec City, Québec, Canada

**Keywords:** Raman spectroscopy, wavelength-swept, swept-source Raman spectroscopy, tissue identification, photon detection

## Abstract

**Significance:**

Raman spectroscopy is a valuable technique for tissue identification, but its conventional implementation is hindered by low efficiency due to scattering. Addressing this limitation, we are further developing the wavelength-swept Raman spectroscopy approach.

**Aim:**

We aim to enhance Raman signal detection by employing a laser capable of sweeping over a wide wavelength range to sequentially excite tissue with different wavelengths, paired with a photodetector featuring a fixed narrow-bandpass filter for collecting the Raman signal at a specific wavelength.

**Approach:**

We experimentally validate our technique using a fiber-based swept-source Raman spectroscopy setup. In addition, simulations are conducted to assess the efficacy of our approach in comparison with conventional spectrometer-based Raman spectroscopy.

**Results:**

Our simulations reveal that the wavelength-swept configuration leads to a significantly stronger signal compared with conventional spectrometer-based Raman spectroscopy. Experimentally, our setup demonstrates an improvement of at least 200× in photon detection compared with the spectrometer-based setup. Furthermore, data acquired from different regions of a fixed monkey brain using our technique achieves 99% accuracy in classification via k-nearest neighbor analysis.

**Conclusions:**

Our study showcases the potential of wavelength-swept Raman spectroscopy for tissue identification, particularly in highly scattering media, such as the brain. The developed technique offers enhanced signal detection capabilities, paving the way for future *in vivo* applications in tissue characterization.

## 1 Introduction

Raman scattering is a phenomenon in which light is inelastically scattered by a material in a way that shifts the frequency of the light to different wavelengths. This shift in frequency can be used to determine the vibrational modes of the material and is therefore a powerful tool for chemical analysis and material characterization.^1^^,^^2^ In Raman spectroscopy, a laser beam is used to excite the sample molecules to a virtual higher energy state. The excited molecules then re-emit light with a different energy when they return to a vibrational excited state, and the difference in frequency between the excitation and scattered light is used to identify chemical bonds and the structure of the sample.^3^^–^^5^ In Raman spectroscopy, the basic setup typically includes a monochromatic light source, usually in the visible or near-infrared range; a sample holder; and a spectrometer as the detector.^3^

Raman spectroscopy can be used to study a wide range of materials, including liquids, gases, and solids. In the field of biophotonics, this technique is of particular interest for biochemical and biological tissue analysis. It has been used to study the microstructure of the tissue, such as the proteins, nucleic acid, and lipids,^6^^–^^8^ as well as the macrostructure of various tissues, such as epiderma or cancer.^9^^,^^10^

Due to the rare nature of Raman scattering events and the presence of noise and background signals, maximizing the collection efficiency of Raman spectroscopy is crucial.^11^ In biological tissues, the presence of an endogenous fluorescence signal is one limiting factor in analyzing the Raman signal because it adds to the photon noise, the variation in measured signal due to the random arrival of photons at a detector, with a broad spectral background, possibly several times more intense than the Raman signal.^12^ Using an IR source allows for reducing the fluorescence but at the expense of the number of Raman photons, which varies in $\lambda^{-4}$.^13^

There are several techniques, such as coherent anti-stokes Raman scattering (CARS) spectroscopy, Fourier transform Raman spectroscopy, and surface-enhanced Raman spectroscopy (SERS), that have been developed to enhance the number of collected Raman photons either by enhancing the Raman scattering at the sample or by improving the detected number of photons.^11^^,^^14^ However, they can be expensive, technically challenging, and less robust compared with the conventional Raman spectroscopy technique.

Nevertheless, the poor collection efficiency due to the presence of a spectrometer in the setup remains the limiting factor for achieving a high signal-to-noise ratio (SNR). The collection efficiency in a spectrometer depends on the entrance slit size and the numerical aperture (NA), which means that increasing the size of the entrance slit and the NA allows more photons to be detected, but this will come at the expense of a lower spectral resolution. In addition, biological tissue exhibits a pronounced tendency to scatter light due to its complex and heterogeneous composition. This scattering phenomenon can significantly impede the effective collection of optical signals. To address this challenge and ensure reasonable collection efficiency, it is imperative to employ a detection system with a substantially expanded optical invariant. The increased optical invariant enables the system to capture and harness the scattered photons, compensating for the scattering effects within biological tissue and thus enhancing the reliability and accuracy of our measurements. It is important to highlight that we are aiming to gather data for spectral identification from a large volume of tissue, not for high spatial resolution imaging.

Many optical imaging and spectroscopy methods benefit from using a wavelength-swept illumination source. Using a wavelength-swept source in optical coherence tomography (OCT) can provide faster and high-resolution data acquisition.^15^ In addition, it increases the SNR 500 times compared with time-domain OCT that uses a non-swept source.^16^ CARS can also be done with a wavelength-swept source to improve the acquisition speed, spectral resolution, and collection efficiency, as we demonstrated in a prior work.^17^ More recently, spontaneous Raman spectroscopy has been performed using a low power wavelength-swept source to increase the photon collection and reduce the noise around 2×.^18^^,^^19^ Replacing the fixed laser in Raman spectroscopy with a swept-source can be advantageous,^20^ and using a wide-area detector enables enhanced signal collection, supporting remote spectrum acquisition for various applications, such as water quality monitoring and indoor farm plant growth tracking.^21^ In addition, replacing the spectrometers with a detector facilitates the development of handheld and miniaturized Raman spectroscopy setups, and it yields the same spectrum as using a conventional Raman spectrometer.^22^ The use of swept-source Raman spectroscopy (SSRS) has also been studied on biological samples, and it can be used to successfully differentiate fat and protein of pork.^23^

In this work, first we present a fiber-based SSRS system and demonstrate that this configuration significantly enhances the photon collection efficiency in thick tissue, yielding an experimental improvement of at least 200 times more photons, as well as increased sensitivity in detection compared with the conventional spectrometer-based technique. We test the setup on non-human primate brain tissue and identify brain regions using the principal component analysis (PCA) with a k-nearest neighbor (KNN) classifier. Finally, we explain why SSRS outperforms a dispersive spectrometer in thick tissue with comprehensive simulations and experiments.

## 2 Methods and Materials

### 2.1 Optical Setups to Obtain Raman Spectra

The wavelength-swept source is a Ti:Sapphire MIRA 900 (Coherent) pumped by a 532 nm Verdi G (Coherent) laser. The Mira configuration was modified to operate the laser in continuous wave (CW) in the auxiliary cavity without dispersion compensation. In addition, the screw knob of the birefringent filter (0163-800-50, Coherent) inside the laser was replaced with a stepper motor actuator (ZST206, Thorlabs) connected to a k-cube stepper motor controller (KST101, Thorlabs) to automatically tune the excitation wavelengths from 800 to 820 nm. The birefringent filter was replaced by the low bandwidth model (3-PLATE BRF Ti:S, Coherent). The narrowband (picosecond) birefringent filter is important for achieving high spectral resolution in the output of the laser. The bandwidth of the excitation wavelength is 0.92 nm in the range of interest. The resolution of the SSRS system is defined by the convolution of the bandwidth of the laser and the narrow-bandpass filter. A bifurcated fiber bundle (BFY600HS02, Thorlabs) with 600  μm core and NA of 0.39 is used to deliver the laser light (power of 150 mW at the sample) and to collect the scattered light from the sample. An ultra-narrow bandpass filter 1064/1 nm (Alluxa) and a low-bandwidth InGaAs femtowatt photoreceiver (Model 2153, Newport) are employed for signal collection, covering the Raman shift region from 2800 to $3100\;{\mathrm{cm}}^{-1}$. This range provides valuable information about the molecular vibration of lipids, ${\mathrm{CH}}_{2}$, and ${\mathrm{CH}}_{3}$ bonds, offering insight into the composition of the cell membrane and myelin in the brain. It is worth mentioning that utilizing the narrowband filter in front of a large area detector with a large input NA will support a much larger invariant and, hence, will detect a beam with a much larger *étendue*. Note that the narrowband interference filter not only has a maximum input cone angle (2 deg or 0.04 NA) but also has a very large diameter of 25 mm. This means that the beam can be spatially expanded (10×) and collimated after the fiber to reduce its solid angle by 10× and enable it to perform as expected.

**Fig. 1:**
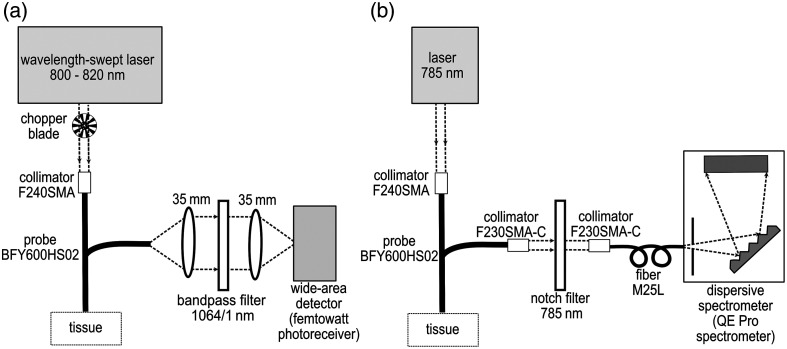
(a) Schematic of the wavelength-swept source setup. The swept-source is a Ti:Sapphire MIRA 900 (Coherent). The laser is chopped and injected into the probe (BFY600HS02, Thorlabs) with a collimator (F240SMA, Thorlabs). The collected light passes through a 4f system (two lenses with a focal length of 35 mm) and an ultra-narrow bandpass filter 1064/1 nm (Alluxa) in-between, and reaches a femtowatt photoreceiver (Model 2153, Newport). (b) Schematic of the dispersive Raman spectrometer setup. A 785 nm CW laser (W785-100FS, Pavillon Integration Technology) is injected into the probe (BFY600HS02, Thorlabs) with a collimator (F240SMA, Thorlabs). Two similar collimators (F240SMA-C, Thorlabs) are used to collimate the laser beam that passes through a notch stop filter at 785 nm, and to inject the signal to a fiber that is connected to the spectrometer (QE Pro, Ocean Insight).

The photoreceiver is connected to a lock-in amplifier (SR830, Stanford Research System) to enable phase-locked detection, thus increasing the SNR. Two primary parameters significantly influence the acquired Raman signal from the lock-in amplifier. The first one is the time constant of the lock-in amplifier. As expected, increasing the time constant of the lock-in amplifier improves the SNR of the acquired spectrum but increases the settling time. To ensure high signal quality without unduly extending the data acquisition duration, we employed a 300 ms time constant for signal recording in our study. The second variable to be set is the chopper frequency. We evaluated the effect of the chopper frequency on the SNR of the signal. Our observations revealed a gradual improvement in the SNR as the chopper frequency increased. However, the SNR exhibited a decline once the chopping frequency exceeded 325 Hz. Given that the detector’s bandwidth extends to 750 Hz, in accordance with the Nyquist rate and the objective of maximizing the SNR, we opted for a chopper frequency of 320 Hz. A homemade program in Python was employed to manage the stepper motor position (wavelength tuning) and the acquisition from the lock-in amplifier. The setup is shown in Fig. 1(a).

For verification purposes, we also built a conventional Raman spectroscopy setup using a 785 nm CW laser (W785-100FS, Pavillon Integration Technology) and the same fiber bundle for light delivery and collection (BFY600HS02, Thorlabs). Using an identical fiber bundle for both excitation and collection in the two setups facilitates a direct comparison of the collected photons at the wide-area detector and the dispersive spectrometer. This approach ensures consistency in photon collection at the sample as the diameter and NA of the collection pathway remain unchanged. The laser power at the sample was 57 mW. A highly sensitive Raman spectrometer (QE Pro, Ocean Insight) with a 50  μm slit (spectral resolution of 0.71 nm) was used for acquiring the spectra. Figure 1(b) shows the configuration of the spectrometer-based setup. The spectra acquired here are used as references for the wavelength-swept system, and their absolute intensities are compared.

**Fig. 2:**
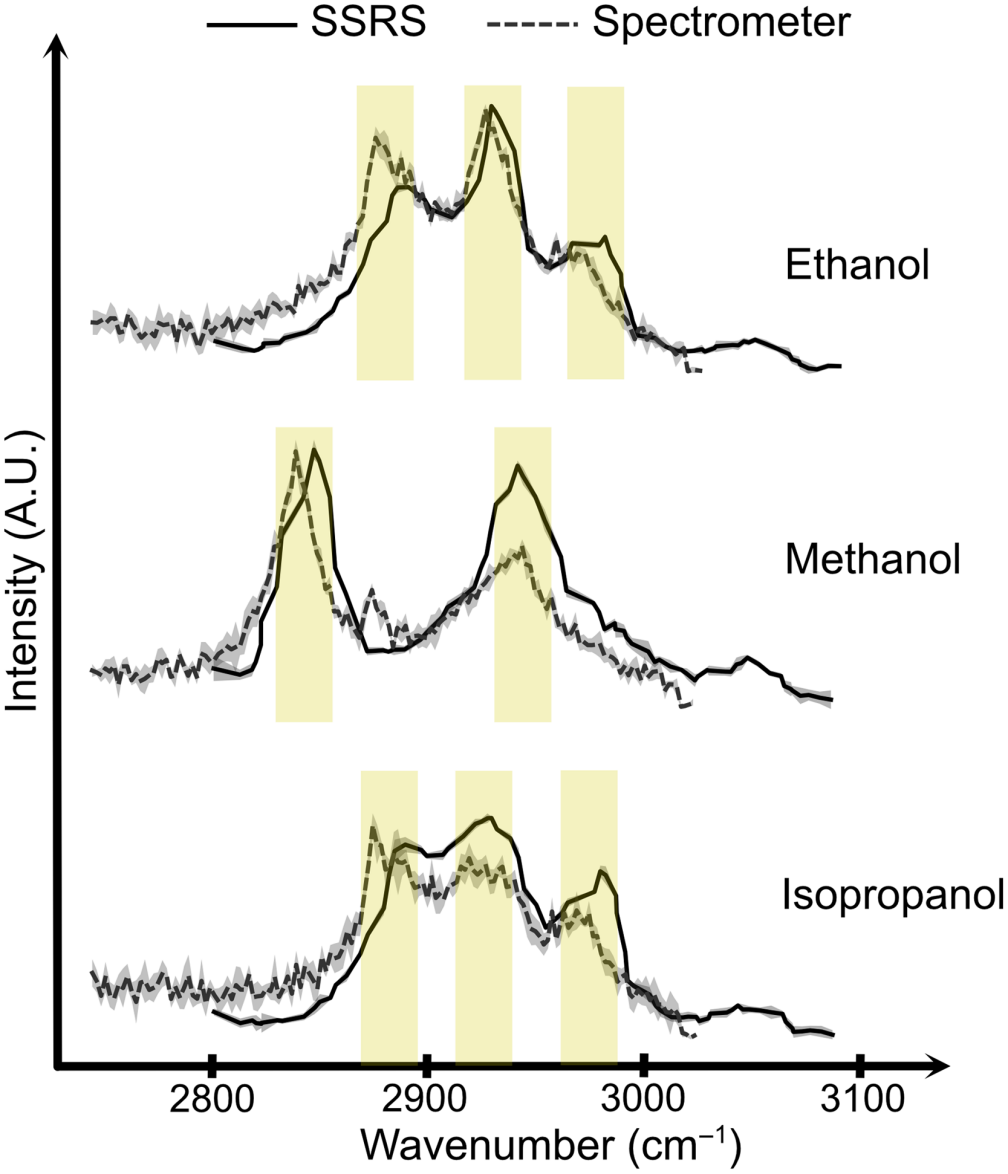
Spectra obtained with the two setups from ethanol, methanol, and isopropanol. The calibration is confirmed by having the Raman peaks at the same wavenumber with the two setups.

Calibration is confirmed with the established Raman spectra (ethanol, methanol, and isopropanol) and is shown in Fig. 2. The peak generated in the ethanol spectrum at $2887\;{\mathrm{cm}}^{-1}$ is the superposition of ${\mathrm{CH}}_{3}$ and ${\mathrm{CH}}_{2}$ symmetric stretches. The other peak at $2934\;{\mathrm{cm}}^{-1}$ is generated by asymmetric ${\mathrm{CH}}_{2}$ bonds, and the last peak at $2975\;{\mathrm{cm}}^{-1}$ is generated by asymmetric ${\mathrm{CH}}_{3}$ bonds.^24^ The two peaks of methanol at 2840 and $2950\;{\mathrm{cm}}^{-1}$ are generated by ${\mathrm{CH}}_{3}$ symmetrical and asymmetrical stretching vibrations, respectively.^24^ The peaks of isopropanol are expected to be at the same wavenumbers as the peaks of ethanol,^25^ and the center peak is expected to be at $2923\;{\mathrm{cm}}^{-1}$. The expected peaks for ethanol, methanol, and isopropanol mentioned in different references and the observed peaks in our graph are presented in Table 1.

**Table 1:** Wavenumber (${\mathrm{cm}}^{-1}$) of expected peaks in the literature and approximate observed peaks in this study for ethanol, methanol, and isopropanol.

Peaks assignment	Data from literature	Observed peaks
Methanol CH sym. stretching	2832^26^, 2834^27^, 2838^28^, 2835^29^	2840
Isopropanol ${\mathrm{CH}}_{3}$ sym. stretching	2879^30^, 2873^31^	2880
Ethanol superposition of ${\mathrm{CH}}_{2}$ and ${\mathrm{CH}}_{3}$ sym. stretching	2887^24^, 2882^32^, 2884^33^, 2880^34^	2880
Ethanol ${\mathrm{CH}}_{2}$ asym. stretching	2934^24^, 2938^32^, 2932^33^, 2929^34^	2930
Isopropanol ${\mathrm{CH}}_{3}$ Fermi resonance	2923^35^, 2928^30^, 2931^31^	2930
Methanol CH asym. stretching	2940^26^, 2943^27^, 2946^28^, 2943^29^	2940
Ethanol ${\mathrm{CH}}_{3}$ asym. stretching	2975^24^, 2983^32^, 2985^33^, 2974^34^	2980
Isopropanol ${\mathrm{CH}}_{3}$ asym. stretching	2980^35^, 2968^30^, 2963^31^	2980

The results shown in Fig. 2 were obtained using the SSRS and spectrometer-based setup. Ideally, these setups should yield an equivalent Raman spectral fingerprint that match the expected peaks in the literature. However, minor discrepancies are observed between the results from the two setups and the reference data. The peaks for the acquired Raman spectra are around the expected wavelength, but the results from the two setups are slightly different ($<10\;{\mathrm{cm}}^{-1}$). We should note that, when measuring with different instruments, a slight difference could be expected.^36^ This might be caused by several factors. The calibration of the spectrometer can affect the accuracy of wavelength scale. The spectral resolution can also cause a slightly shifted Raman spectra. Finally, the samples are nominally the same, but there might be a small difference in the concentration or morphology.

We measured the Raman spectra of pure methanol, gray matter (GM), and white matter (WM) samples to compare the number of detected photons using the SSRS setup and spectrometer-based setup. The three samples were chosen with different scattering properties to study the effect of scattering on the detected photons. Each acquisition was repeated five times to average and increase the SNR. The acquisitions with the spectrometer were done with a laser power of 57 mW at the sample and an integration time of 5 s. The spectra units are in counts; with a well depth of 300,000 and 16 bits, one count is equal to eight photons. In addition, the dark noise was measured to be subtracted from the measured Raman spectra. To make the SSRS spectra comparable with the ones acquired with the spectrometer-based setup, we need to convert the measured spectra units from volts to a number of photons, which is calculated as

$$P_{\text{in}}=\frac{V_{\text{out}}}{R\cdot G},$$ (1)

where R is the detector responsivity (A/W), corresponding to 0.75 at 1064 nm, and G is the transimpedance gain, equal to $2\times 10^{10}\;V/A$.^37^ The input power of the detector ($P_{\text{in}}$) is interpreted as the energy of the photons reaching the detector per second. In addition, the energy of the photons is converted to the number of photons using E=hc/λ, where h is Plank’s constant equal to $6.63\times 10^{-34}\;J\cdot s$, c is the light speed ($3\times 10^{8}\;m/s$), and λ is the wavelength of the detected photon, which is 1064 nm in our case. Another important factor to be considered for comparing the two setups is the laser power at the sample. Because the Raman signal is linearly proportional to the laser power, we assume that the SSRS setup will generate ∼2.5× more Raman photons than the spectrometer-based setup. Although the illumination sources in both systems are at different wavelengths, we do not consider the Raman efficiency to be significantly affected because $(\lambda_{\text{excitation}}/785{)}^{4}∼1.13$, or nearly 1.^38^^–^^40^ In addition, we neglect the effect of the swept-source power change. The 150 mW is the maximum power at around 810 nm, and there is a decrease of the maximum by 10% at 800 and 820 nm where there is no Raman peak in the spectra.

### 2.2 Brain Tissue Preparation

The brain sample is from a non-human primate (*Macaca fascicularis*). The monkey was sacrificed, and the whole brain was extracted and fixed by immersion in paraformaldehyde 4%. Brain slices of 1 mm were obtained using vibratome and collected serially in phosphate-buffered saline (PBS).

### 2.3 Brain Tissue Classification Using the SSRS Spectra

Using the 1 mm-thick brain slices, we acquired 140 spectra, 20 from each region, including WM and regions with GM, such as subthalamic nucleus (STN), the internal (GPi) and external (GPe) globus pallidus, substantia nigra (SN), and striatum (putamen and caudate nucleus). The brain regions were identified by a neuroanatomist using a stereotaxic atlas of *Macaca fascicularis* brain.^41^ The classification was performed with a KNN on the features obtained from PCA with leave-one-out cross-validation in Python using the *scikit-learn* package.

## 3 Results

**Fig. 3:**
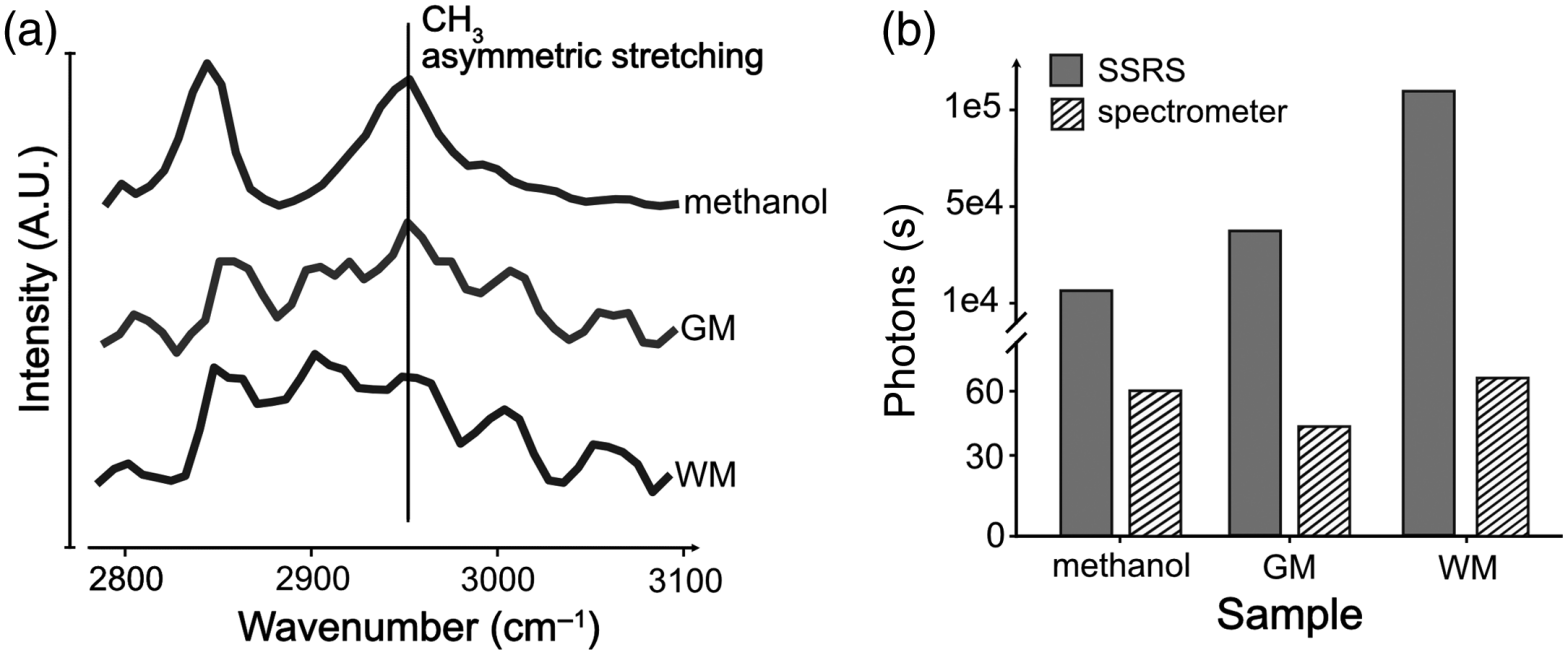
Experimental comparison of the Raman spectra of three different samples: methanol, GM, and WM. (a) Raman spectra obtained from three samples with different scattering properties (methanol < GM < WM) for the SSRS setup. (b) The average number of collected Raman photons in five acquisitions for each sample at $2951\;{\mathrm{cm}}^{-1}$, which is a peak caused by ${\mathrm{CH}}_{3}$ asymmetric stretching for both setups. The number of photons is per second of acquisition time. It shows the increased signal for the SSRS system as scattering increases, contrary to the spectrometer-based system in which the signal stays mostly constant.

The measured spectra from several samples (WM, GM, and methanol) are plotted in Fig. 3(a) for the SSRS setup. The three samples are chosen with different scattering properties. Methanol exhibits lower light scattering properties compared with GM, and both methanol and GM have lower light scattering characteristics than WM. The ${\mathrm{CH}}_{3}$ asymmetric stretching vibration peak that is observed in the methanol Raman spectrum^24^ is also generated in WM and GM. Other peaks in WM and GM at 2845 and $2920\;{\mathrm{cm}}^{-1}$ are generated by lipids ${\mathrm{CH}}_{2}$ and ${\mathrm{CH}}_{3}$ bonds.^42^

In Fig. 3(b), we compare the average number of photons in five acquisitions that are collected at $2951\;{\mathrm{cm}}^{-1}$, which is the peak for ${\mathrm{CH}}_{3}$ asymmetric stretching. The dispersive spectrometer collects around 10,000 times less photons compared with the SSRS. Even after correcting for the total integration time (45 s for SSRS versus 1 s for dispersive spectrometer), the dispersive spectrometer is still more than 200 times less efficient than SSRS. Furthermore, the graphical representation, Fig. 3(b), illustrates that, as scattering intensifies, there is a corresponding augmentation in the photon count of the SSRS with no change observed in the dispersive spectrometer. This indicates that the wavelength-swept system performs better as the scattering increases, which makes it the ideal strategy for characterizing biological tissues.

**Fig. 4:**
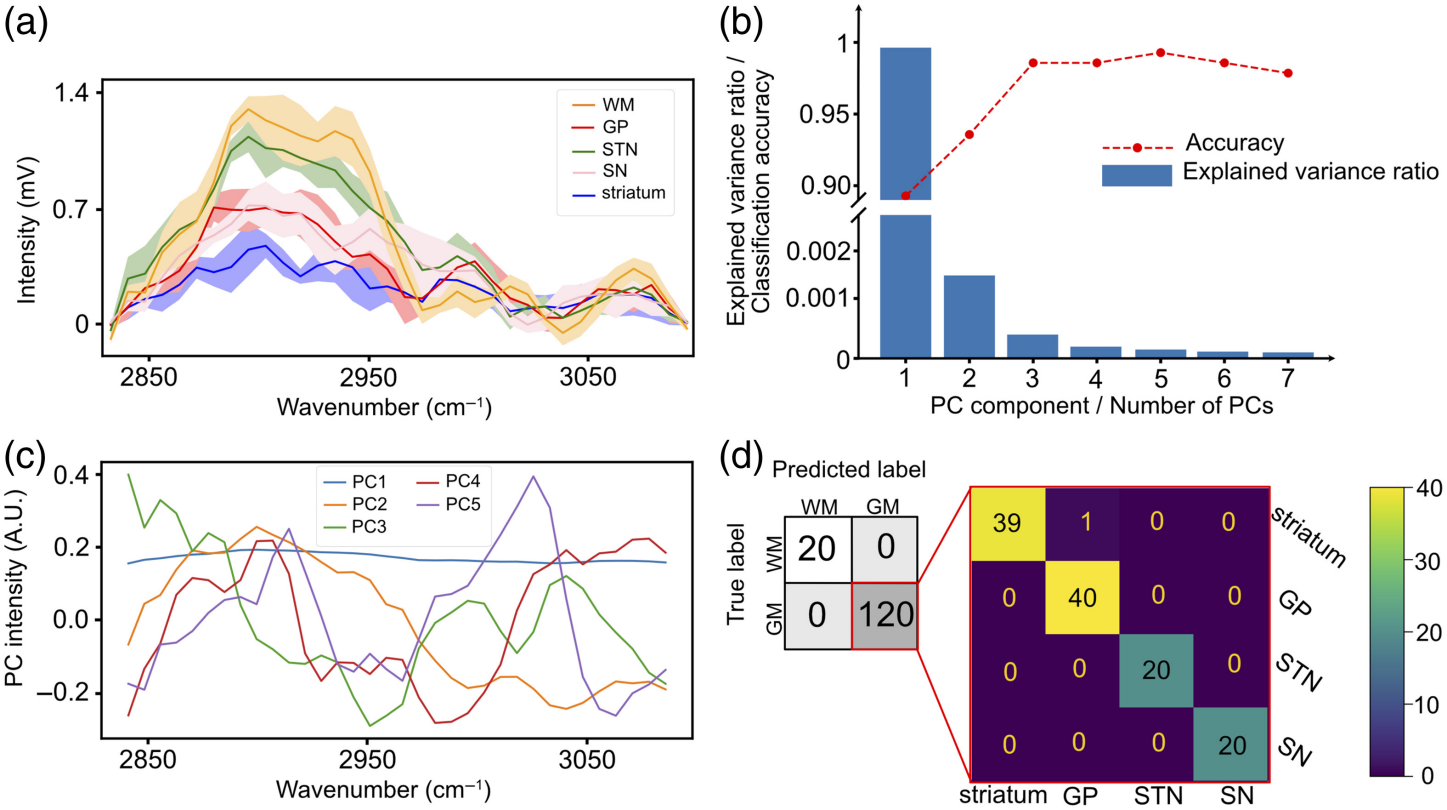
(a) Spectra of different brain regions obtained with the SSRS system. The baseline of the spectra is removed to make them comparable. (b) Explained variance ratio of each principal component and the accuracy of the classification using KNN. (c) The first five principal components that are used for classification. (d) Confusion matrix of brain regions classification using five first principal components. The accuracy of the classification is 99.2%.

We therefore attempted to classify brain regions based on their Raman spectra [shown in Fig. 4 (a)]. The most important component from PCA is the first principal component (PC1), which explains 99% of the variation of the data. However, the accuracy of the classification still improves as we include up to five PCs [Fig. 4(b)]. Classification using leave-one-out with the first five PCs gives a 99.2% accuracy. The confusion matrix of the classification [Fig. 4(d)] shows that the classification accuracy of GM and WM is 100%. In addition, regions containing GM, such as the striatum, globus pallidus, SN, and STN, are well separated with a KNN classifier looking at three nearest neighbors. The first five PCs that are used for classification are shown in Fig 4(c). This indicates that PC1 helps differentiate WM and GM, but the last four PCs are necessary for differentiating the various types of GM.

## 4 Discussion

First, our results show that, based on the classification results obtained with SSRS data, we can distinguish distinct types of GM using the Raman spectra obtained with the SSRS setup. By looking at the first five PCs, we can find the peaks at different wavelengths in the high wavenumbers that are used to differentiate several regions in the brain. The peaks at these wavenumbers mostly correspond to lipids and carbohydrates. The caudate nucleus and the putamen together form the striatum in primates. Both of these regions are largely composed of the same cellular and molecular elements and could be distinguished from the two segments of the pallidum (GPe and GPi) that also share similar molecular and cellular composition. The experiment was performed on fixed tissue that is more stable and less fragile than fresh tissue. The results of the classification presented here and obtained from fixed brain tissue do not translate directly to fresh tissue as there might be some other Raman features introduced by the fixation process,^43^ but a similar strategy could definitely be used.

As shown in Fig. 3(b), the SSRS system acquires spectra and performs well in scattering tissues, apparently better than a spectrometer-based system. This warrants a more in-depth investigation as it may come as a surprise. What is the main aspect of this system that makes it so good at collecting light from scattering media? When attempting to collect light with a large *étendue*, the optical invariant of the system is critical in determining the detection efficiency. We demonstrate both computationally and experimentally that the SSRS system has a much larger optical invariant and thus is much more efficient with wide diffuse sources than its spectrometer-based counterpart.

A numerical comparison of the detection efficiency between SSRS and conventional Raman spectroscopy has been done using the Raytracing Python module.^44^ The total efficiency in the two setups includes the collection efficiency at the sample and the coupling efficiency at the detector. In biological tissues, light scattering results in a large scattering cone, encompassing a wide range of directions (i.e., a large *étendue*). The collection efficiency of the system will need to have a correspondingly large optical invariant to collect with minimal losses (i.e., large NA and large fiber diameter). The coupling efficiency refers to the proportion of light rays emitted from the fiber, successfully traversing the lenses and filters, and being detected at the detector or the spectrometer. To estimate the total efficiency, we simulated the scattering source as a two-dimensional (2D) disk with a uniform distribution having a diameter of 0.5 mm and NA of 0.5. The fiber was simulated using an aperture with a defined NA and diameter. The 4f system composed of two 35 mm lenses of Fig. 1 with magnification M=1 that is used to concentrate the light on the area of the detector or the entrance slit of the spectrometer is considered to be lossless, so they can be disregarded in the simulations. At the end of the optical path, an aperture with a diameter of 1 mm and NA of 0.4, as the acceptance cone of the photodetector, was placed to represent the area of the detector. In the second optical path, the aperture width was set to 50  μm with a NA of 0.1, and the aperture height was set to 500  μm with a NA of 0.25 to simulate the slit of the spectrometer. The parameters used in the simulations, including the diameter and NA of both the wide-area detector and the dispersive spectrometer, correspond to the specifications of the detectors utilized in the experimental setup in Figs. 1(a) and 1(b).

**Fig. 5:**
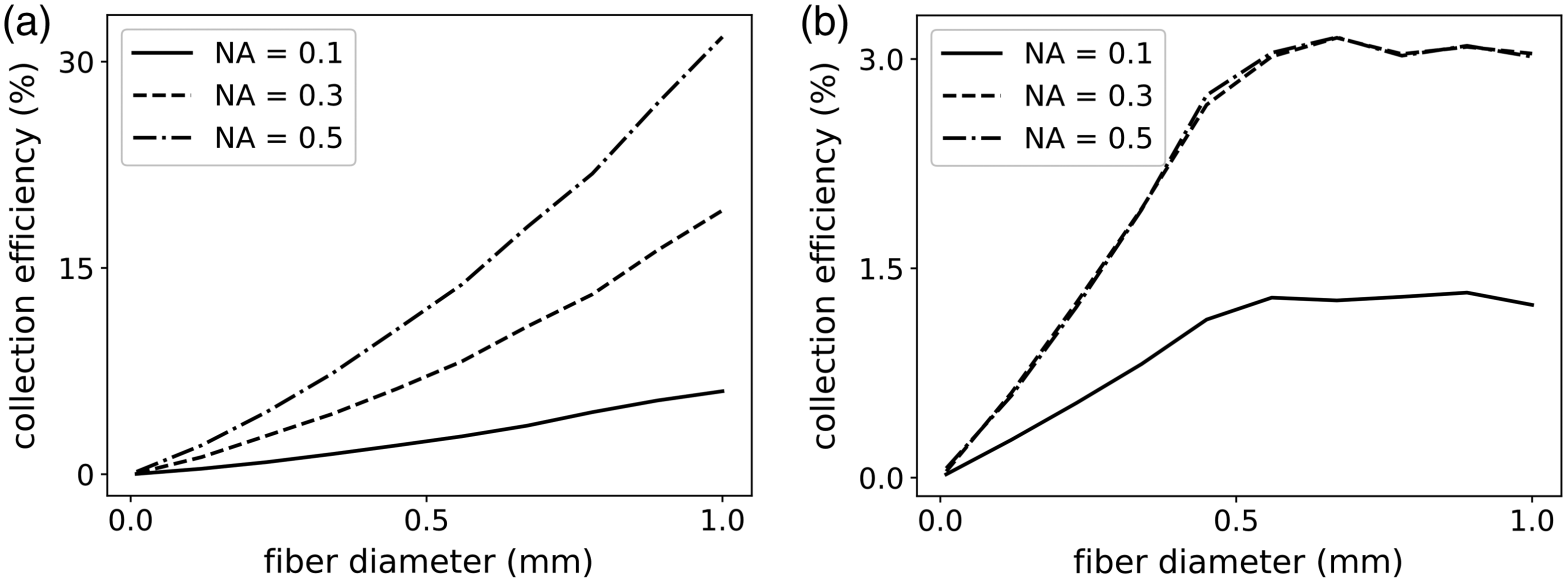
Collection efficiency of (a) the SSRS setup and (b) spectrometer-based setup using 100,000 rays as the Raman scattering at the sample (a 2D disk with a uniform distribution having a diameter of 0.5 mm and NA of 0.5). The collection fiber NA varies from 0.1 to 0.5, and the fiber diameter varies from 0.01 to 1 mm. Note that the y-axis scale of the SSRS plot is 10 times larger than that of the spectrometer-based setup.

The results of the simulation of the efficiency by changing a fiber with a diameter and NA for collection at the sample in the two setups are presented in Figs. 5(a) and 5(b) and show that the highest collection efficiency of the spectrometer-based setup is around 3%, whereas it reaches 30% in the SSRS setup. The collection efficiency increases as the optical invariant of the fiber is increased in the SSRS configuration, but not in the dispersive spectrometer. This is explained by their respective optical invariants: the wide-area detector has a large optical invariant, supporting the detection of a large *étendue* beam, but the dispersive spectrometer has a significantly lower optical invariant, which renders useless any improvements on the fiber side. This therefore suggests using an optical fiber with an optical invariant essentially equal to the invariant of the detection configuration.

**Fig. 6:**
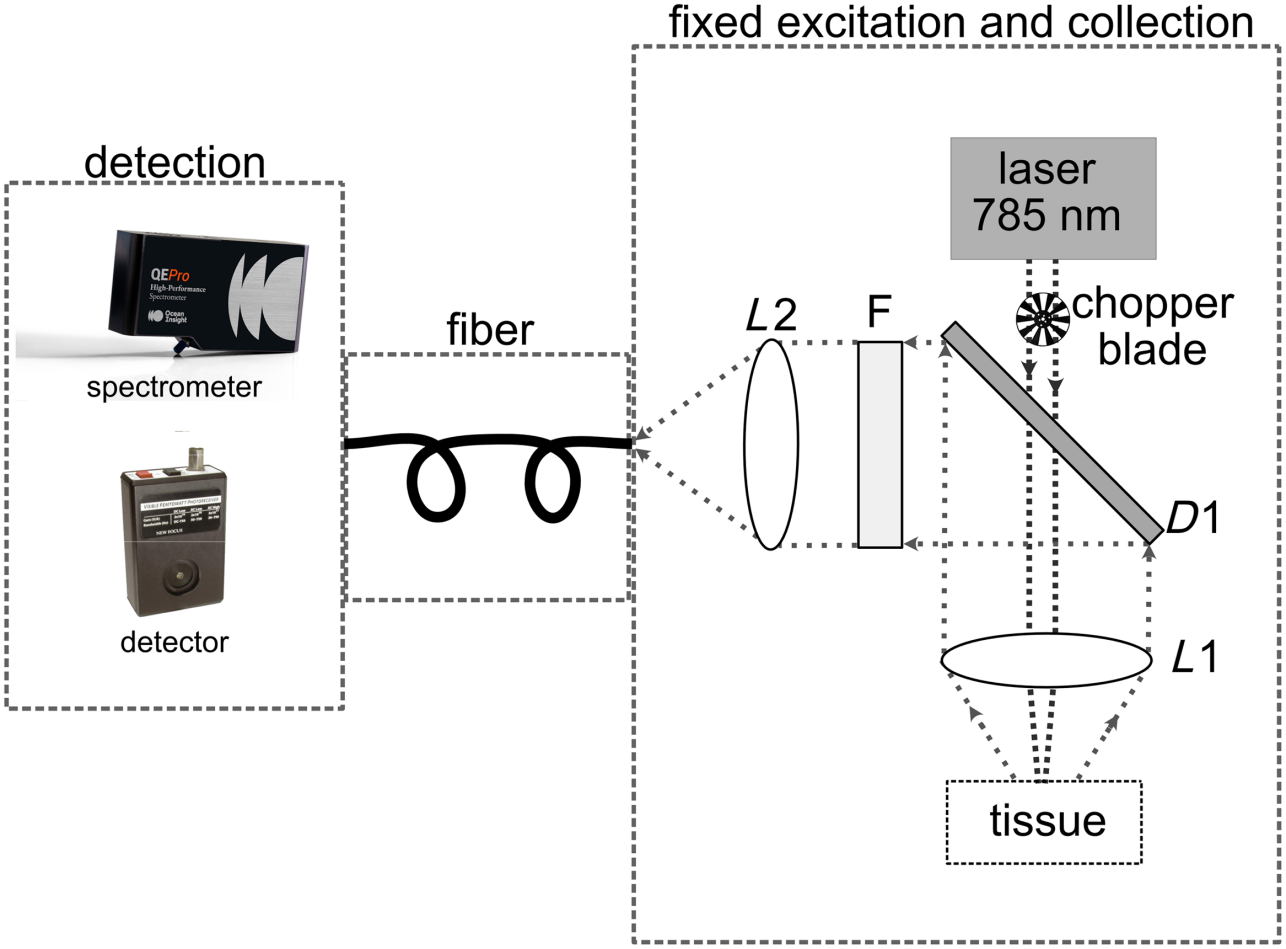
Schematic of the simplified setup with a common illumination and collection, but two different collection schemes: one with a dispersive spectrometer and another with a wide detector. The spectrometer has an SMA fiber connector and for the wide-area detector; we used an SMA fiber adapter to connect the fiber directly into the detector. The laser (785 nm CW -W785-100FS, Pavilion Integration Technology) passes through D1 (shortpass @ 800 nm, Edmund Optics). The focal lengths of L1 and L2 are 17.5 and 50 mm, respectively. With the wide-area detector (InGaAs femtowatt photoreceiver—2153, Newport), F1 is an ultra-narrow bandpass filter 1064/1 nm (Alluxa). When using the spectrometer (QE Pro, Ocean Insight), F1 is a notch filter at 785 nm (OD 4.0 @ 785 nm, Edmund Optics). The fiber is varied in the experiment to compare the efficiency based on different optical invariants of the fiber: M96L01, M92L01, M45L01, M53L01, M59L01, and M93L01—Thorlabs.

To confirm this experimentally, we conducted a systematic comparison with a simplified setup but two different collection schemes. Using the same excitation and collection optics but using a dispersive spectrometer (QE Pro, Ocean Insight—with a 50  μm slit) or a wide-area detector (InGaAs femtowatt photoreceiver—2153, Newport), we compare their respective collection efficiencies. The setup is shown in Fig. 6. The setup starts with a common 785 nm CW laser (W785-100FS, Pavilion Integration Technology); the laser power at the sample was 57 mW. The diameter of the lenses and filters is 25 mm. The focal lengths of L1 and L2 are 17.5 and 50 mm, respectively. A dichroic (shortpass @ 800 nm, Edmund Optics) is used to transmit the laser beam and reflect the Raman light scattering that has been collected from the tissue. The beam then goes through a filter F, dependent on the detection scheme: when measuring with the spectrometer, F is a notch filter at 785 nm (Edmund Optics, OD 4.0 @ 785 nm) to prevent the spectrometer from being saturated. On the other hand, when measuring with the wide-area detector, the filter F is an ultra-narrow bandpass filter 1064/1 nm (Alluxa) to detect only a specific Raman wavenumber. In addition, the chopper blade connected to the lock-in amplifier is also placed right after the laser when using the wide-area detector, and it is removed when we use the spectrometer. Then, the beam is focused into various optical fibers with distinct diameters ($d_{\mathrm{of}}$) and NAs, thereby possessing different optical invariants, calculated simply with $\mathrm{NA}\times d_{\mathrm{of}}$. The fibers under consideration exhibited optical invariants of 10.5, 44, 200, 288, 500, and 585  μm (M96L01, M92L01, M45L01, M53L01, M59L01, and M93L01—Thorlabs). The investigation used WM and GM from a calf fixed brain and a set of fibers with different characteristics (diameter and NA). We obtained 10 spectra with each fiber from WM and GM.

**Fig. 7:**
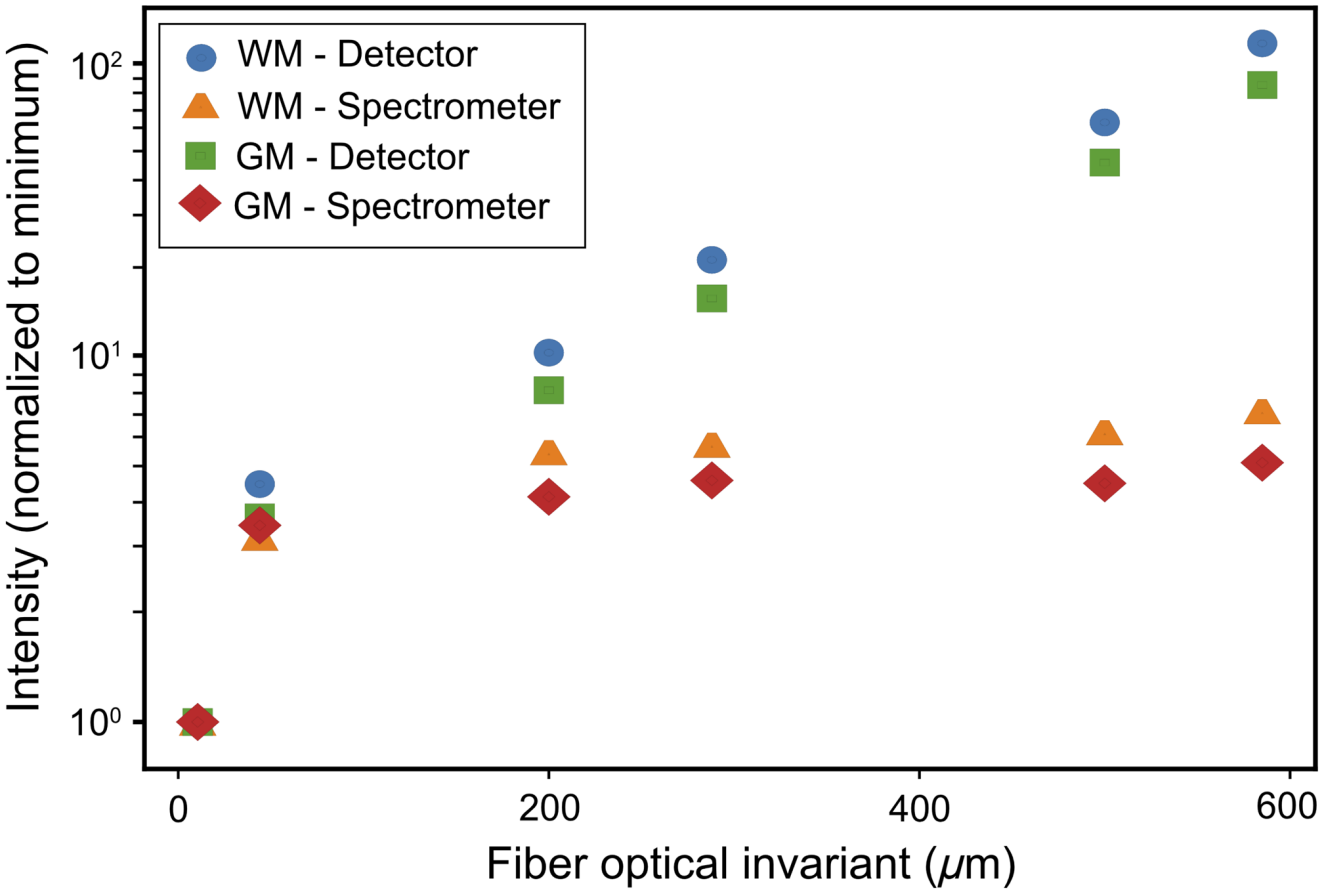
Increase in the ratio of the intensity of the peaks in WM and GM obtained with the same setup but with two different collection systems (spectrometer versus detector). The y-axis is shown in log scale. We can see that, as the invariant of the collection increases, the collection of the detector outperforms the collection of the spectrometer.

The findings regarding the comparison of signal intensity using various fibers are depicted in Fig. 7. The intensity of the peak at $600\;{\mathrm{cm}}^{-1}$ was employed for plotting data obtained with the spectrometer, whereas the value acquired for the detector corresponds to the water peak at $3340\;{\mathrm{cm}}^{-1}$. To enhance the comparability between the two collection methods, spectrometer and detector, all values obtained by each detection system were normalized by the minimum value, namely, the value acquired by the fiber with the smallest optical invariant. This normalization procedure ensures a standardized basis for assessing and contrasting the signal intensities across the different detection systems.

We can conclude from Fig. 7, as we use larger fibers with a higher NA, that the collection efficiency increases with the fiber optical invariant, but the collection in the dispersive spectrometer is only slightly increasing because the invariant of the spectrometer is already limiting the detection. On the other hand, the wide-area detector that can accept a large cone of light can significantly increase the light collection from the tissue. The dispersive spectrometer has a slit size of 50  μm and NA of around 0.1 (optical invariant of 5  μm), and the wide-area detector has a detection area of $1\;{\mathrm{mm}}^{2}$ and NA of ∼0.4 (optical invariant of 400  μm). When comparing, the wide-area detector’s invariant is 80 times greater than that of the dispersive spectrometer. Consequently, this allows for a significantly higher photon collection capability when utilizing a larger fiber for coupling the collected photons into the detection hardware. Consequently, the larger optical invariant of the wide-area detector is a fundamental factor in circumventing the challenges posed by biological samples that are highly scattering, and increasing the optical invariant of the fiber is beneficial, as long as it does not surpass the optical invariant of the detection scheme.

According to Figs. 7 and 5, in which we compared the collection of the wide-area detector with the dispersive spectrometer, we can observe that there is a slight increase in the efficiency of the dispersive spectrometer when using a fiber with a higher optical invariant. This elevation arises because the entrance slit of the spectrometer is a 2D iris, and the width and NA of the entrance slit of the spectrometer are limited as discussed, but the height of the slit is not limited. Therefore, increasing the diameter and the NA of the fiber will affect the collection due to the height of the slit. In spectroscopy with a dispersive spectrometer, one approach to increasing the number of collected photons is to use fiber bundles. However, the charge-coupled devices (CCDs) used in the spectrometers are limited in height. In our case, the height is 58 pixels, each 24  μm (∼1.3  mm), which is comparable to the height of the wide-area detector. Moreover, the light emerging from the fiber diverges as it passes through optical elements to reach the CCD array. Consequently, the beam’s height expands, and not all photons can be detected by the detector, even if the height of our fiber bundle matches that of the CCD array. Therefore, despite the wide-area detector and dispersive spectrometers having comparable sizes, the substantially larger width of the detector provides us with a significant advantage.

Due to the higher number of photons that has been detected in SSRS, we can expect to have a higher SNR (at least √200∼15 times higher). In a spectrometer, sources of noise include readout noise, dark current shot noise, and photon noise. To enhance the quality of measurements, one can consider strategies, such as prolonging acquisition times, employing high-intensity lasers, and utilizing detectors with minimal noise characteristics. In the SSRS setup, employing a battery-powered detector minimizes noise to the greatest extent possible. To further study the sources of the noise, we obtained 28 spectra of methanol and calculated the standard deviation of the spectra at each wavenumber. The shape of the standard deviation is not correlated with the number of photons acquired for each data point as expected by the shape of the noise spectra in the manual of the detector. In other words, the noise level remains constant and exhibits small random fluctuations regardless of whether the signal is weak or strong. This can be an advantageous characteristic in some situations because it implies that the noise does not worsen as the signal becomes weaker. In such cases, the noise remains relatively constant, which can make it easier to isolate the noise and detect low-intensity signals.

The SNR also benefits from using a lock-in amplifier for narrow-band detection that can increase the SNR significantly. The thermal noise and the shot noise in a photoreceiver are modeled as

$$N_{\text{Noise}}=\frac{4\mathrm{BW}K_{b}T}{R}+\frac{2BW\eta e^{2}P_{\text{sample}}}{E_{v}}\;[A^{2}],$$ (2)

where e is the photon elementary charge, $E_{v}$ is the photon energy, $P_{\text{sample}}$ is the sample power, $K_{b}$ is the Boltzmann’s constant, T is the temperature in degrees Kelvin, R is the value of the transimpedance amplifier feedback resistor, and BW is the detection bandwidth. We can see that the noise is directly related to the bandwidth of the detection, and with a lock-in amplifier, we are limiting the detection bandwidth and reducing the noise from the photoreceiver.

## 5 Conclusion

Our study presented a wavelength-swept Raman spectroscopy (SSRS) strategy that addresses the efficiency limitations of conventional Raman spectroscopy, particularly in highly scattering tissues. This cost-effective alternative to techniques such as CARS and SERS utilizes a laser that sweeps over a broad wavelength range, coupled with a photodetector employing a fixed narrow-bandpass filter. The method offers a large optical invariant as a key practical advantage, confirmed by simulations and experiments. The technique extends its application to brain tissue analysis, particularly in distinguishing different regions. Classification results based on KNN showcase the promising performance of SSRS in identifying distinct types of GM, providing valuable insights into brain tissue composition.

In summary, our research establishes SSRS as a reliable and efficient method for Raman spectroscopy, offering simplicity and enhanced performance, especially in highly scattering tissues. Future investigations should explore the broader implications of the SSRS setup, particularly in advancing *in vivo* acquisitions and its application in various biomedical research contexts.

## Data Availability

Code and data underlying the results presented in this paper are not publicly available at this time but may be obtained from the authors upon request.
